# Supplementary material for: Tailored design of protein nanoparticle scaffolds for multivalent presentation of viral glycoprotein antigens
Source: eLife. 2020 Aug 4;9:e57659. doi: 10.7554/eLife.57659 (PMC7402677; doi:10.7554/eLife.57659)
Supplement: Figure 2—source data 1. — Experimentally-measured data (exp) is compared to predicted design data (model). Molecular weights (MW) were obtained using the ASTRA software. Rg and Dmax calculations performed in Scatter3 SAXS analysis software with the determined qmax values. X values computed from the FoXS online SAXS web server between the designed model and the experimental scattering data. [file elife-57659-fig2-data1.docx]

| **Design** | MW A (kDa) | MW B (kDa) | MW model (kDa) | MW exp (kDa) | Rg model (Å) | Rg exp (Å) | Dmax model (Å) | Dmax exp (Å) | *X* | qmax (1/nm) |
| --- | --- | --- | --- | --- | --- | --- | --- | --- | --- | --- |
| **1na0C3_2** | 14.99 | - | 44.97 | 48 | 26.4 | 29.5 | 84 | 86 | 1.4 | 0.23 |
| **3ltjC3_1v2** | 20.90 | - | 62.69 | 56 | 27.7 | 31.3 | 88 | 94 | 1.1 | 0.18 |
| **3ltjC3_11** | 22.21 | - | 66.62 | 50 | 28.3 | 30.3 | 87 | 92 | 1.6 | 0.20 |
| **HR04C3_5v2** | 23.05 | - | 69.14 | 71 | 25.9 | 28.6 | 82 | 86 | 1.5 | 0.25 |
|  |  |  |  |  |  |  |  |  |  |  |
| **T33_dn2** | 13.82 | 14.88 | 344.45 | 397 | 61.4 | 64.7 | 169 | 169 | 4.8 | 0.17 |
| **T33_dn5** | 13.72 | 21.49 | 422.42 | 422 | 66.7 | 69.4 | 177 | 193 | 1.7 | 0.16 |
| **T33_dn10** | 14.07 | 31.42 | 545.88 | 556 | 62.3 | 60.1 | 169 | 170 | 2.3 | 0.20 |
| **O43_dn18** | 22.69 | 13.82 | 876.26 | 810 | 80.6 | 81.3 | 217 | 221 | 2.9 | 0.28 |
| **I53_dn5** | 17.19 | 15.33 | 1951.57 | 2000 | 95.9 | 97.1 | 241 | 243 | 1.2 | 0.21 |

**Figure 2-Source Data 1.** **Biophysical properties of designed trimers and two-component nanoparticles**. Experimentally-measured data (exp) is compared to predicted design data (model). Molecular weights (MW) were obtained using the ASTRA software. R_g_ and D_max_ calculations performed in Scatter3 SAXS analysis software with the determined q_max_ values. *X* values computed from the FoXS online SAXS web server between the designed model and the experimental scattering data.
