# Supplementary material for: Tailored design of protein nanoparticle scaffolds for multivalent presentation of viral glycoprotein antigens
Source: eLife. 2020 Aug 4;9:e57659. doi: 10.7554/eLife.57659 (PMC7402677; doi:10.7554/eLife.57659)
Supplement: Figure 2—figure supplement 1—source data 1. [file elife-57659-fig2-figsupp1-data1.docx]

| Design | Target Molecular Weight (kDa) | Experimental Molecular Weight (kDa) | Approximate Oligomerization State |
| --- | --- | --- | --- |
| 1na0C3_1 | 44 | 43 | 3 |
| 2fo7C3_1 | 51 | 20 | 1 |
| 2fo7C3_12 | 50 | 106 | 6 |
| 2fo7C3_15 | 51 | 70 | 4 |
| 3ltjC3_1 | 63 | 57 | 3 |
| HR10C3_7 | 67 | 47 | 2 |
| HR10C3_7v2 | 67 | 38 | 2 |
| HR10C3_18 | 67 | 35 | 2 |
| HR4C3_1 | 69 | 75 | 3 |
| HR4C3_5 | 69 | 61 | 3 |
| HR4C3_7 | 68 | 65 | 3 |
| HR7C3_9 | 56 | 30 | 2 |
| tpr1C3_6 | 48 | 28 | 2 |
| HR00C3_3 | 94 | 37 | 1 |

**Figure 2-figure supplement 1-source data 1.** **SEC-MALS data for off-target designed trimers.**
