# Supplementary material for: Tailored design of protein nanoparticle scaffolds for multivalent presentation of viral glycoprotein antigens
Source: eLife. 2020 Aug 4;9:e57659. doi: 10.7554/eLife.57659 (PMC7402677; doi:10.7554/eLife.57659)
Supplement: Figure 2—figure supplement 3—source data 1. — Statistics for the highest-resolution shell are shown in parentheses. [file elife-57659-fig2-figsupp3-data1.docx]

|  | **1na0C3_2 (PDB ID X)** | **3ltjC3_1v2 (PDB ID Y)** |
| --- | --- | --- |
| **Data Acquisition** |  |  |
| Space group | P 1 21 1 | R 3 :H |
| Cell dimensions  a, b, c (Å)  α , β , γ (°) | 69.47, 64.91, 99.03  90, 106.33, 90 | 88.182, 88.182, 65.244  90, 90, 120 |
| R_merge_ | 0.1427 (0.8023) | 0.1189 (1.041) |
| CC_1/2_ | 0.994 | 0.997 (0.623) |
| <I/σ_I_> | 9.5 (2.16) | 9.35 (1.30) |
| Completeness (%) | 99.86 | 91.51 (77.88) |
| Multiplicity | 4.6 (4.6) | 5.6 (5.1) |
| Wilson B-factor (Å^2^) | 37.11 | 40.43 |
| **Refinement** |  |  |
| Resolution range (Å) | 38.87 - 2.53 (2.60 - 2.53) | 44.09 - 2.303 (2.386 - 2.303) |
| No. of reflections for refinement | 27066 (2030) | 8361 (655) |
| No. of reflections for R_free_ | 1442 (103) | 756 (62) |
| R_work_(%)/R_free_(%) | 0.1859 (0.195) / 0.2316 (0.240) | 0.2021 (0.2555) / 0.2261 (0.2240) |
| Water count | 110 | 25 |
| Residue count | 711 | 180 |
| Average B-factors (Å^2^)  Protein  Water | 47.40  46.01 | 49.36  50.80 |
| r.m.s.d. deviations  Bond length (Å)  Bond angles (°) | 0.68  0.78 | 0.002  0.44 |
| Ramachandran favored (%) | 98.00 | 100.00 |
| Ramachandran allowed (%) | 1.00 | 0.00 |
| Ramachandran outliers (%) | 1.00 | 0.00 |
| Rotamer outliers (%) | 3.00 | 0.00 |

**Figure 2-figure supplement 3-Source Data 1.** **Crystallography data collection and refinement statistics for designed trimers 1na0C3_2 and 3ltjC3_1v2.** Statistics for the highest-resolution shell are shown in parentheses.
