# Supplementary material for: Tailored design of protein nanoparticle scaffolds for multivalent presentation of viral glycoprotein antigens
Source: eLife. 2020 Aug 4;9:e57659. doi: 10.7554/eLife.57659 (PMC7402677; doi:10.7554/eLife.57659)
Supplement: Table 1—source data 1. [file elife-57659-table1-data1.docx]

| Design | Tailored Antigens | Experimental Molecular Weight (kDa) | Target Molecular Weight (kDa) | SAXS *X* value | Resolution, backbone r.m.s.d.  structure (Å, Å) |
| --- | --- | --- | --- | --- | --- |
| 1na0C3_2 | HA, SOSIP, DS-Cav1 | 48 | 45 | 1.4 | 2.6, 1.4 |
| 3ltjC3_1v2 | SOSIP, DS-Cav1 | 56 | 63 | 1.1 | 2.3, 0.8 |
| 3ltjC3_11 | SOSIP, DS-Cav1 | 50 | 66 | 1.6 | -- |
| HR04C3_5v2 | SOSIP | 71 | 69 | 1.5 | -- |
| T33_dn2 | HA, SOSIP, DS-Cav1 | 397 | 345 | 4.8 | -- |
| T33_dn5 | HA, SOSIP, DS-Cav1 | 422 | 422 | 1.7 | -- |
| T33_dn10 | HA, SOSIP, DS-Cav1 | 546 | 556 | 2.3 | 3.9, 0.65 |
| O43_dn18 | HA,SOSIP, DS-Cav1 | 810 | 876 | 2.9 | 4.5, 0.98 |
| I53_dn5 | HA, SOSIP, DS-Cav1 | 2000 | 1960 | 1.2 | 5.3, 1.30 |

**Table 1.** **Summary of the experimental characterization for designed trimers and two-component nanoparticles.** 1na0C3_2 and 3ltjC3_1v2 structures determined by X-ray crystallography and T33_dn10, O43_dn18, and I53_dn5 structures determined by cryo-EM.
