# Supplementary material for: Tailored design of protein nanoparticle scaffolds for multivalent presentation of viral glycoprotein antigens
Source: eLife. 2020 Aug 4;9:e57659. doi: 10.7554/eLife.57659 (PMC7402677; doi:10.7554/eLife.57659)
Supplement: Figure 4—source data 1. [file elife-57659-fig4-data1.docx]

|  | **T33_dn10** | **O43_dn18** | **I53_dn5** |
| --- | --- | --- | --- |
| Microscope | Titan Krios | Titan Krios | Titan Krios |
| Voltage (kV) | 300 | 300 | 300 |
| Detector | Gatan K2 Summit | Gatan K2 Summit | Gatan K2 Summit |
| Recording mode | Counting | Counting | Counting |
| Magnification | 29,000 X | 29,000 X | 29,000 X |
| Movie micrograph pixel size | 1.03 | 1.03 | 1.03 |
| Dose rate (e^−^/Å^2^/s) | 5.04 | 5.04 | 4.46 |
| No. of frames per movie micrograph | 40 | 40 | 45 |
| Frame exposure time (ms) | 250 | 250 | 250 |
| Movie micrograph exposure time (s) | 10.00 | 10.00 | 11.25 |
| Total dose (e^−^/Å^2^) | 50.4 | 50.4 | 50.2 |
| Under focus range (µm) | 0.6 - 1.6 | 0.6 - 1.6 | 0.6 - 1.6 |
| Number of movie micrographs | 502 | 1,336 | 1,548 |

**Figure 3-Source Data 1.** **Cryo-EM data acquisition metrics for designed nanoparticles T33_dn10, O43_dn18, and I53_dn5.**
