# Supplementary material for: Tailored design of protein nanoparticle scaffolds for multivalent presentation of viral glycoprotein antigens
Source: eLife. 2020 Aug 4;9:e57659. doi: 10.7554/eLife.57659 (PMC7402677; doi:10.7554/eLife.57659)
Supplement: Figure 4—source data 2. [file elife-57659-fig4-data2.docx]

|  | **T33_dn10** | **O43_dn18** | **I53_dn5** |
| --- | --- | --- | --- |
| PDB | 6VFH | 6VFI | 6VFJ |
| Residues | 4,752 | 7,560 | 16,320 |
| Amino-acids | 4,752 | 7,560 | 16,320 |
| Carbohydrates | 0 | 0 | 0 |
| RMSD Bonds | 0.019 | 0.018 | 0.020 |
| RMSD Angles | 1.389 | 1.484 | 1.648 |
| Ramachandran |  |  |  |
| Favored (%) | 99.13 | 98.71 | 98.88 |
| Allowed (%) | 0.87 | 1.29 | 1.12 |
| Outliers (%) | 0.00 | 0.00 | 0.00 |
| Rotamer outliers | 0.00 | 0.00 | 0.00 |
| Clash score | 0.32 | 0.41 | 0.27 |
| Molprobity score | 0.62 | 0.65 | 0.60 |
| EMRinger score | 2.12 | 0.68 | 0.69 |

**Figure 3-Source Data 2.** **Cryo-EM model building and refinement statistics for designed nanoparticles T33_dn10, O43_dn18, and I53_dn5.**
