# Supplementary material for: Tailored design of protein nanoparticle scaffolds for multivalent presentation of viral glycoprotein antigens
Source: eLife. 2020 Aug 4;9:e57659. doi: 10.7554/eLife.57659 (PMC7402677; doi:10.7554/eLife.57659)
Supplement: Supplementary file 1. — (A) Amino acid sequences for all designed trimers and de novo homo-oligomers used for two-component nanoparticle design. Sequences include initiating methionines and His6-tags. Designed trimers that expressed solubly are denoted in bold, and experimental methods used for characterization are included in parentheses. *Components from previously described designed homo-oligomers in Fallas et al., 2017 or the Protein Data Bank (PDB ID). (B) Amino acid sequences for all designed two-component nanoparticles. Sequences include initiating methionines and His6-tags. Designs that expressed solubly and co-eluted from IMAC are denoted in bold. Input oligomers from (A) are included in parentheses. (C) Amino acid sequences for all antigen-fused trimeric nanoparticle components. Sequences include initiating methionines and signal peptides. [file elife-57659-supp1.docx]

| Design | Sequence |
| --- | --- |
| **1na0C3_1**  **(SEC-MALS)** | **MNIAEAAYRVGNKAYKKGRYELAILAYILAILLDPNNAEAWYNLGNAYYKEGEYDEAIEYYQKALELDPNNAEAWYNLGNAYYKQGDYDEAIEYYQKALELDPNNAEAKQNLGNAKQKQGLEHHHHHH** |
| **1na0C3_2**  **(SEC-MALS, SAXS)** | **MEEAELAYLLGELAYKLGEYRIAIRAYRIALKRDPNNAEAWYNLGNAYYKQGDYDEAIEYYQKALELDPNNAEAWYNLGNAYYKQGDYDEAIEYYQKALELDPNNAEAKQNLGNAKQKQGLEHHHHHH** |
| **2fo7C3_1**  **(SEC-MALS)** | **MAERLYKLGNKAYKRGEYILALIAYVVALRDDPRSAEAWYNLGNAAYKSGEYDEAIEYYQKALELDPRSAEAWYNLGNAYYKQGDYDEAIEYYQKALELDPRSAEAWYNLGNAYYKQGDYDEAIEYYQKALELDPRSLEHHHHHH** |
| **2fo7C3_12**  **(SEC-MALS)** | **MAEKAYNIGNAAYKEGEYRVAILAYMLALLADPRSAEALYNLGNAAYKEGDYKVAIAAYLLALDLDPRSAEAWYNLGNAYYKQGDYDEAIEYYQKALELDPRSAEAWYNLGNAYYKQGDYDEAIEYYQKALELDPRSLEHHHHHH** |
| **2fo7C3_15**  **(SEC-MALS)** | **MALRWLLLGILAMLLGAEELAIEAYQKALELEPRSAMAWLALGAAYYKEGDYDEAIEYYQKALELDPRSAAAWALLGNAYYKQGDYDEAIEYYQKALENRPRSARAWYNLGNAYYKQGDYDEAIEYYQKALELDPRSLEHHHHHH** |
| **3ltjC3_1**  **(SEC-MALS)** | **MTDPLAVILYIAILKAEKSIARAKAAEALGKIGDERAVEPLIKALKDEDALVRAAAADALGQIGDERAVEPLIKALKDEEGLVRASAAIALGQIGDERAVEPLIKALKDERDLVRVAAAVALGRIGDERAVEPLIKALKDEEGEVREAAAIALGSIGGERVRAAMEKLAETGTGFARKVAVNYLETHKLEHHHHHH** |
| **3ltjC3_1v2**  **(SEC-MALS, SAXS)** | **MTDPMKVILYIAMLELEKYIMRAAAAYALGKIGDERAVEPLIKALKDEDAIVRAAAADALGQIGDERAVEPLIKALKDEDGAVRVSAAVALGQIGDERAVEPLIKALKDEDAVVRVAAAIALGLIGDERAVEPLIKALKDEKGKVREAAALALGAIGGERVRAAMEKLAETGTGFARKVAVNYLETHKLEHHHHHH** |
| **3ltjC3_11**  **(SEC-MALS, SAXS)** | **MRREETDPLAVVMYRLNLRDDSYYVRRAAAYALGKIGDERAVEPLIKALKDEDAWVRRAAADALGQIGDERAVEPLIKALKDEDGWVRQSAAVALGQIGDERAVEPLIKALKDEDWFVRAAAAAALGRIGDERAVEPLIKALKDEDEMVREIAALALGMIGGERVRAAMEKLAETGTGFARKVAVNYLETHKLEHHHHHH** |
| **HR4C3_1**  **(SEC-MALS)** | **MDICELEARLVALLVLLAKRAGADEDLIAELVAVMIMIVILRLKKSGSSYEVICECVARIVAEIVEALKRSGTSEDEIAEIVARVISEVIRALKRSGSSYEVICECVARIVAEIVEALKRSGTSEDEIAEIVARVISEVIRTLKESGSSYEVIKECVQRIVEEIVEALKRSGTSEDEINEIVRRVKSEVERTLKESGSLEHHHHHH** |
| **HR4C3_5**  **(SEC-MALS)** | **MDECEEKARRVAEKVERLKRSGTSEDEIAEEVAREISEVIRTLKESGSEYKVICRCVARIVAEIVEALKRSGTSEDEIAEIVARVISEVIRTLKESGSKYKIICICVAIIVAEIVAALKRSGTSEDEIAEIVARVISEVIRTLKESGSSYEVIKQCVQAIVQAIILALMKSGTEVEEILLIVLRVEEEVERTLKESGSLEHHHHHH** |
| **HR4C3_5v2**  **(SEC-MALS, SAXS)** | **MDECEEKARRVAEKVERLKRSGTSEDEIAEEVAREISEVIRTLKESGSEYKVICRCVARIVAEIVEALKRSGTSEDEIAEIVARVISEVIRTLKESGSDYLIICVCVAIIVAEIVEALKRSGTSEDEIAEIVARVISEVIRTLKESGSSYEVIKECVQIIVLAIILALMKSGTEVEEILLILLRVKTEVRRTLKESGSLEHHHHHH** |
| **HR4C3_7**  **(SEC-MALS)** | **MDECEKKARLVAILVIVAKALGAEEKLIALLVALEIVVVIIELKASGSSYEVICECVARIVAEIVEALKRSGTSEDEIAEIVAKVIAAVIIVLKELGSSYEVICECVARIVAEIVEALKRSGTSEDEIAEIVARVISEVIRTLKESGSSYEVIKECVQRIVEEIVEALKRSGTSEDEINEIVRRVKSEVERTLKESGSLEHHHHHH** |
| **HR7C3_2**  **(SEC)** | **MEKRIARELCELAAERAAESNDEREARIAAIECLLVAERAGMPTKEAARSFCEAAARAAAESNDEEVAKIAAKACLEVAKQAGMPTKEAARSFCEAAARAAAESNDEEVAKIAAKACLEVAKQAGMPTKEAARSFCEAAKRAAKESNDEEVEKIAKKACKEVAKQAGMPLEHHHHHH** |
| HR7C3_8 | MTEEDAARTCKKAARKAAESNDEEVAKQAAKDCLEVAKQAGMPTTIAAAIFCLAAARAAAESNDEEVAKIAAKACLEVAKQAGMPTKAAAIAFCIAAAMAAAESRDEEVAKIAAKACLEVAKQAGMPTKTAAALFMIAAIAAALRSEDEVVLAIAALAIAEVLKQAGMPLEHHHHHH |
| **HR7C3_9**  **(SEC-MALS)** | **MTKEMAAVLCMVLALKAAESNDEEKAKKAAKLCLIMADEAGMPTKEAARSFCEAAAIAAAVSEDEEVAKIAAKACLEVAKQAGMPTKEAARSFCEAAASAAAILNEEEVAKIAAKACLEVAKQAGMPTKEAARSFCEAAKRAAKRSNDEEVEKIAKKACKEVAKQAGMPLEHHHHHH** |
| **HR10C3_7**  **(SEC-MALS)** | **MSSEKEELRELLVAIVAVAAEDKGDDTEEAREAAREAFELVREAAERAGIDSSEVLTLAILLILIVVLIAADAGYDISEAARAAAEAFKRVAEAAKRAGITSSEVLELAIRLIKEVVVNAAIRGYDISEAARAAAEAFKRVAEAAKRAGITSSKILKMAIILIRVMVKMAKERGKDISEAARQAAEIFRKAAERMRGSLEHHHHHH** |
| **HR10C3_7v2**  **(SEC-MALS)** | **MSSEKEELRKMLVALVVVAAKEKGDDTEEAREAAREAFELVREAAERAGIDSSVVLALAILLILLVVLAAQMAGYDISEAARAAAEAFKRVAEAAKRAGITSSEVLELAIRLIKRVVLNAQIRGYDISEAARAAAEAFKRVAEAAKRAGITSSLLLKMAIVLIRVLVELAQESGADISEAARKAAEIMRRAAEDMRGSLEHHHHHH** |
| **HR10C3_8**  **(SEC)** | **MDECEEKARRVAEKVERLKRSGTSEDEIAEEVAREISEVIRTLKESGSEEVEICACVARIVAEIVEALKRSGTSEDEIAEIVARVISEVIRTLKESGSSYLVICMCVALIVAQIVEALKRSGTSRKEIAEIVARVISEVIRTLKESGSSYEVIKECVERIVRAIVLALRESGTRITEIMAIVLAVLKEVLRTLKESGSLEHHHHHH** |
| **HR10C3_18**  **(SEC-MALS)** | **MKREKMELAKRLLKIVVENAKRKGDEEALAALAALLAFALVREAAERAGIDSSEVLELAIRLIKEVVENAQREGYRIALAALVAAMAFAVVAEAAKEAGITSSEVLELAIRLIKEVVENAQREGYEIVDAAMAAALAFARVAEAAKRAGITSSETLKRAIEEIRKRVEEAQREGNDISEAAEQAAEEFRKKAEELKLEHHHHHH** |
| **HR00C3**  **(SEC)** | **MKEEKIAKLISLLAELSKKLIEIVARAADNKTTEEAVDIAILLIAIIARLAIRLIEMLAKNLASEEFMARAISAIAELAKKAIEAIYRLADNHTTDIRMLKAILAIAELAAEAIKAIADLAKNHTTEEFMARAISAIAELAKKAIEAIYRLADNHTTDLFMAIAIMAIAVLALLAIMAIADLAKNHTTEEFMAKAISAIAELAKKAIEAIYRLADNHTSPDLIELAILAIEIIALAAIIAIEELAENITTEEYKEKAKSAIDEIREKAKEAIKRLEDNRTLEHHHHHH** |
| **HR00C3_3**  **(SEC-MALS)** | **MKERLIAKLISVLAEASKILIRIAAKAADKLEREAAVILAIVLIAVAAIAAIAAIALLAANLASEEFMARAISAIAELAKKAIEAIYRLADNHTEDEAMALAIEIIAILALLAIVAIALLAANHTTEEFMARAISAIAELAKKAIEAIYRLADNHTTDTFMAKAIEAIAELAKEAIKAIAELAKNHTTEEFMAKAISAIAELAKKAIEAIYRLADNHTSPTYIEKAIEAIEKIARTAIKAIEDLAKNITTEEYKEKAKSAIDEIREKAKEAIKRLEDNRTLEHHHHHH** |
| **HR00C3_44**  **(SEC)** | **MTEEKIAKEISRIAEESKKRIEELARKADNKTREAVVALAIAKIALLAREAIKRIEDLAKNLASEEFMARAISAIAELAKKAIEAIYRLADNHTKDVLMLVAIVAIAELAKEAIKAIADLAKNHTTEEFMARAISAIAELAKKAIEAIYRLADNHRLVAAMLLAIEAIAELAKEAIKAIADLAKNHTTEEFMAKAISAIAELAKKAIEAIYRLADNHRLPAAILLAALAILLIAVTAILAILILALNITTEEYKEKALSAIEEIVEKAEEAIDRLEDNLTLEHHHHHH** |
| **tpr1C3_6**  **(SEC-MALS)** | **MAEAWKELGKVLEKLGRLEEAAVAYLLAVIDDPNDAEAWKELGKVLEKLGELDAAAVAYEAAIELDPNDAEAWKELGKVLEKLGRLRRAALAYIKAIALDPNDAEAWKELGKVAEKLGRLKIAARIYKKAIELDPNDLEHHHHHH** |
| **ank1C2_1*** | **MHHHHHHGSWGSSELGKRLIEAAENGNKDRVKDLIENGADVNASDSDGRTPLHHAAENGHAEVVALLIEKGADVNAKDSDGRTPLHHAAENGHDEVVLILLLKGADVNAKDSDGRTPLHHAAENGHKRVVLVLILAGADVNTSDSDGRTPLDLAREHGNEEVVKALEKQ** |
| **ank3C2_1*** | **MSELGKRLIEAAENGNKDRVKDLLENGADVNASDSDGKTPLHLAAENGHAKVVLLLLEQGADPNAKDSDGKTPLHLAAENGHAVVVALLLMHGADPNAKDSDGKTPLHLAAENGHEEVVILLLAMGADPNTSDSDGRTPLDLAREHGNEEVVKVLEDHGGWLEHHHHHH** |
| **1na0C3_3*** | **MNLAEKMYKAGNAMYRKGQYTIAIIAYTLALLKDPNNAEAWYNLGNAAYKKGEYDEAIEAYQKALELDPNNAEAWYNLGNAYYKQGDYDEAIEYYQKALELDPNNAEAKQNLGNAKQKQGLEHHHHHH** |
| **1na0C3_7*** | **MNSAEAMYKMGNAAYKQGDYILAIIAYLLALEKDPNNAEAWYNLGNAAYKQGDYDEAIEYYQKALELDPNNAEAWYNLGNAYYKQGDYDEAIEYYQKALELDPNNAEAKQNLGNAKQKQGLEHHHHHH** |
| **HR00C3_2*** | **MIEEVVAEMIDILAESSKKSIEELARAADNKTTEKAVAEAIEEIARLATAAIQLIEALAKNLASEEFMARAISAIAELAKKAIEAIYRLADNHTTDTFMARAIAAIANLAVTAILAIAALASNHTTEEFMARAISAIAELAKKAIEAIYRLADNHTTDKFMAAAIEAIALLATLAILAIALLASNHTTEEFMAKAISAIAELAKKAIEAIYRLADNHTSPTYIEKAIEAIEKIARKAIKAIEMLAKNITTEEYKEKAKSAIDEIREKAKEAIKRLEDNRTLEHHHHHH** |
| **1na0C4_1*** | **MTLARVAYILGAIAYAQGEYDIAITAYQVALDLDPNNAEAWYNLGNAYYKQGDYDEAIEYYQKALELDPNNAEAWYNLGNAYYKQGDYDEAIEYYQKALELDPNNAEAKQNLGNAKQKQGLEHHHHHH** |
| **HR04C4_1*** | **MHHHHHHGSWGSDECEEKARRVAEKVERLKRSGTSEDEIAEEVAREISEVIRTLKESGSSYEVICECVARIVAEIVEALKRSGTSAVEIAKIVARVISEVIRTLKESGSSYEVICECVARIVAEIVEALKRSGTSAAIIALIVALVISEVIRTLKESGSSFEVILECVIRIVLEIIEALKRSGTSEQDVMLIVMAVLLVVLATLQLSGS** |
| **2JFB (PDB ID)*** | **MAVKGLGEVDQKYDGSKLRIGILHARWNRKIIDALVAGAVKRLQEFGVKEENIIIETVPGSFELPYGSKLFVEKQKRLGKPLDAIIPIGVLIKGSTMHFEYICDSTTHQLMKLNFELGIPVIFGVLTCLTDEQAEARAGLIEGKMHNHGEDWGAAAVEMATKFN** |
| **2OBX (PDB ID)*** | **MNQHSHKDYETVRIAVVRARWHADIVDQCVSAFEAEMADIGGDRFAVDVFDVPGAYEIPLHARTLAETGRYGAVLGTAFVVNGGIYRHEFVASAVIDGMMNVQLSTGVPVLSAVLTPHNYHDSAEHHRFFFEHFTVKGKEAARACVEILAAREKIAA** |
| **2B98 (PDB ID)*** | **MTKKVGIVDTTFARVDMASIAIKKLKELSPNIKIIRKTVPGIKDLPVACKKLLEEEGCDIVMALGMPGKAEKDKVCAHEASLGLMLAQLMTNKHIIEVFVHEDEAKDDKELDWLAKRRAEEHAENVYYLLFKPEYLTRMAGKGLRQGFEDAGPARE** |

**Supplementary File 1A.** **Amino acid sequences for all designed trimers and *de novo* homo-oligomers used for two-component nanoparticle design.** Sequences include initiating methionines and His_6_-tags. Designed trimers that expressed solubly are denoted in bold, and experimental methods used for characterization are included in parentheses.

*Components from previously described designed homo-oligomers in Fallas et al. (2017) or the Protein Data Bank (PDB ID).

| Design | Sequence |
| --- | --- |
| T33_dn1A  (1na0C3_3)  T33_dn2B  (1na0C3_2) | MGNLAEKMYKAGNAMYRKGQYTIAIIAYTLALLKDPNNAEAWYNLGNAAYKKGEYDEAIEAYQKALELDPNNAEAWYNLGNAYYKQGDYDEAIEYYERALELDPENAEAALNLLEAKEKQG  MEEAELAYLLGELAYKLGEYRIAIRAYRIALKRDPNNAEAWYNLGNAYYKQGDYREAIKYYAKALTLDPKNAEAWYNLGNAVYKQGDYRIAILFYRAALKLDPNNAEAKQNLGNAKQKQGLEHHHHHH |
| **T33_dn2A**  **(1na0C3_3)**  **T33_dn2B**  **(1na0C3_2)** | **MGNLAEKMYKAGNAMYRKGQYTIAIIAYTLALLKDPNNAEAWYNLGNAAYKKGEYDEAIEAYQKALELDPNNAEAWYNLGNAYYKQGDYDEAIEYYKKALRLDPRNVDAIENLIEAEEKQG**  **MEEAELAYLLGELAYKLGEYRIAIRAYRIALKRDPNNAEAWYNLGNAYYKQGDYREAIRYYLRALKLDPENAEAWYNLGNALYKQGKYDLAIIAYQAALEEDPNNAEAKQNLGNAKQKQGLEHHHHHH** |
| T33_dn3A  (1na0C3_7)  T33_dn3B (1na0C3_2) | MGNSAEAMYKMGNAAYKQGDYILAIIAYLLALEKDPNNAEAWYNLGNAAYKQGDYKEAILYYIRALQLDPNNAEAWYNLGNAFYKKGDYRVAIILYRMALKLDPNNAEAKQNLGNAKQKQGDIHHHHHH  MEEAELAYLLGELAYKLGEYRIAIRAYRIALKRDPNNAEAWYNLGNAYYKQGDYDEAIEYYQKALELDPNNAEAWYNLGNAYYKQGDYEEAILYYLEALDLDPNNAEAAENLLNAVKKDE |
| **T33_dn4A**  **(1na0C3_7)**  **T33_dn4B**  **(3ltjC3_1)** | **MGNSAEAMYKMGNAAYKQGDYILAIIAYLLALEKDPNNAEAWYNLGNAAYKQGDYDEAIEYYQKALELDPNNAEAWYNLGNAYYKQGDYDEAIEYYEKALELDPRNAEALKNLLEAKAKQD**  **MHHHHTDPLAVILYIAILKAEKSIARAKAAEALGKIGDERAVEPLIKALKDEDALVRAAAADALGQIGDERAVEPLIKALKDEEGLVRASAAIALGQIGDERAVRPLIKALADERDLVRVAAAVALGRIGDERAVKPLIIVLLDEEGEVREAAAIALGSIGGERVRAAMEKLAERGRGFARKVAVNYLETHKLEHHHHHH** |
| **T33_dn5A**  **1na0C3_7**  **T33_dn5B**  **(3ltjC3_1)** | **MGNSAEAMYKMGNAAYKQGDYILAIIAYLLALEKDPNNAEAWYNLGNAAYKQGDYDEAIEYYQKALELDPNNAEAWYNLGNAYYKQGDYDEAIEYYEKALELDPNNAEALKNLLEAIAEQD**  **MHHHHTDPLAVILYIAILKAEKSIARAKAAEALGKIGDERAVEPLIKALKDEDALVRAAAADALGQIGDERAVEPLIKALKDEEGLVRASAAIALGQIGDERAVQPLIKALTDERDLVRVAAAVALGRIGDEKAVRPLIIVLKDEEGEVREAAAIALGSIGGERVRAAMEKLAERGTGFARKVAVNYLETHKLEHHHHHH** |
| **T33_dn6A**  **(1na0C3_2)**  **T33_dn6B**  **(3ltjC3_1)** | **MGEEAELAYLLGELAYKLGEYRIAIRAYRIALDEDPDNAEAWYNLGNAYYKQGDYREAILYYQMALRLDPNNAEAWYNLGNAYYKQGDYDRAIEYYQKALELDPNNAEAKQNLGNAKQKQGDIHHHHHH**  **MHHHHTDPLAVILYIAILKAEKSIARAKAAEALGKIGDERAVEPLIKALKDEDALVRAAAADALGQIGDERAVEPLIKALKDEEGLVRASAAIALGQIGDKRAVRPLIRALKDERDLVREAAAVALGRIGDELAVEPLIKALKDEEGEVREAAAIALGSIGGEIVRMMMDKLAETGTGFARKVAVNYLETHK** |
| **T33_dn7A**  **(1na0C3_2)**  **T33_dn7B**  **(3ltjC3_1)** | **MGEEAELAYLLGELAYKLGEYRIAIRAYRIALKRDPNNAEAWYNLGNAYYKQGDYDEAIEYYQKALELDPNNAEAWYNLGNAYYKQGDYDEAIEYYRKALELDPENEEALENLLNAKQKQGDIHHHHHH**  **MHHHHTDPLAVILYIAILKAEKSIARAKAAEALGKIGDERAVEPLIKALKDEDALVRAAAADALGQIGDERAVEPLIKALKDEEGLVRASAAIALGQIGDERAVEPLIKALKDERDLVRVAAAVALGRIGDERAVEPLIKALKDEEGEVREAAAIALGSIGGKRVRLAMLKLALEGTGFARKVAVNYLETHK** |
| T33_dn8A  (1na0C3_2)  T33_dn8B  (HR00C3_2) | MGEEAELAYLLGELAYKLGEYRIAIRAYRIALKRDPNNAEAWYNLGNAYYKQGDYDEAIEYYQKALELDPNNAEAWYNLGNAYYKQGDYDEAIEYYRKALELDPENLEALLNLLNAKDKRG  MIEEVVAEMIDILAESSKKSIEELARAADNKTTEKAVAEAIEEIARLATAAIQLIEALAKNLASEEFMARAISAIAELAKKAIEAIYRLADNHTTDTFMARAIAAIANLAVTAILAIAALASNHTTEEFMARAIRAIAELAKKAIEAIYRLADNHTTDKFMAAAIEAIALLATLAILAIALLASNHTTERFMAKAILAIAVLAKKAIEAIYRLADNHTSPTYIEKAIEAIEKIARKAIKAIEMLAKNITTEEYKEEAKSAIEIIRELARIAIRRLEDNRTLEHHHHHH |
| T33_dn9A  (1na0C3_2)  T33_dn9B  (HR00C3_2) | MGEEAELAYLLGELAYKLGEYRIAIRAYRIALKRDPNNAEAWYNLGNAYYKQGDYDEAIEYYQKALELDPNNAEAWYNLGNAYYKQGDYDEAIEYYQKALELDPENLEAILNLGEAKLKQG  MIEEVVAEMIDILAESSKKSIEELARAADNKTTEKAVAEAIEEIARLATAAIQLIEALAKNLASEEFMARAISAIAELAKKAIEAIYRLADNHTTDTFMARAIAAIANLAVTAILAIAALASNHTTEEFMARAISAIAELAKKAIEAIYRLADNHTTDKFMAAAIEAIALLATLAILAIALLASNHTTEKFMAEAIIVIALLAVLAIMAIYRLADNHTSPTYIEKAIEAIEKIARKAIKAIEMLAKNITTEEYKEKAKSAIDLIRQLADIIIRKLEDNRTLEHHHHHH |
| **T33_dn10A**  **(1na0C3_2)**  **T33_dn10B**  **(HR00C3_2)** | **MGEEAELAYLLGELAYKLGEYRIAIRAYRIALKRDPNNAEAWYNLGNAYYKQGDYDEAIEYYQKALELDPNNAEAWYNLGNAYYKQGDYDEAIEYYEKALELDPENLEALQNLLNAMDKQG**  **MIEEVVAEMIDILAESSKKSIEELARAADNKTTEKAVAEAIEEIARLATAAIQLIEALAKNLASEEFMARAISAIAELAKKAIEAIYRLADNHTTDTFMARAIAAIANLAVTAILAIAALASNHTTEEFMARAISAIAELAKKAIEAIYRLADNHTTDKFMAAAIEAIALLATLAILAIALLASNHTTEKFMARAIMAIAILAAKAIEAIYRLADNHTSPTYIEKAIEAIEKIARKAIKAIEMLAKNITTEEYKEKAKKIIDIIRKLAKMAIKKLEDNRTLEHHHHHH** |
| T33_dn11A  (1na0C3_2)  T33_dn11B  (HR00C3_2) | MGEEAELAYLLGELAYKLGEYRIAIRAYRIALKRDPNNAEAWYNLGNAYYKQGDYDEAIEYYQKALELDPNNAEAWYNLGNAYYKQGDYDEAIEYYRKALELDKENIEALLNLLNAKEKQD  MIEEVVAEMIDILAESSKKSIEELARAADNKTTEKAVAEAIEEIARLATAAIQLIEALAKNLASEEFMARAISAIAELAKKAIEAIYRLADNHTTDTFMARAIAAIANLAVTAILAIAALASNHTTEEFMARAISAIAELAKKAIEAIYRLADNHTTDKFMAAAIEAIALLATLAILAIALLASNHTTERFMAKAILAIAILAAKAIEAIYRLADNHTSPTYIEKAIEAIEKIARKAIKAIEMLAKNITTEEYKEEAKSAIEIIRLLAKAVIKRLQDNRTLEHHHHHH |
| **O32_dn1A**  **(1na0C3_3)**  **O32_dn1B**  **(ank1C2_1)** | **MGELAEKMYKAGNAMYRKGQYTIAIIAYTLALLKDPNNAEAWYNLGNAAYKKGEYDEAIVAYVEALELDPNNAEAWYNLGNAYYKQGDYEEAIEYYQKALELDPNNAEAKQNLGNAKQKQG**  **MSRRGRLLIIAAENGNKDRVKDLIQRGADVNASDRRGRTPLHHAAENGHAEVVALLIEKGADVNAKDSDGRTPLHHAAENGHDEVVLILLLKGADVNAKDSDGRTPLHHAAENGHKRVVLVLILAGADVNTSDSDGRTPLDLAREHGNEEVVKALEKQLEHHHHHH** |
| O32_dn2A  (1na0C3_2)  O32_dn2B  (ank1C2_1) | MGEEAELAYLLGELAYKLGEYRIAIRAYRIALKRDPNNAEAWYNLGNAYYKQGDYDEAIEYYQKALELDPNNAEAWYNLGNAYYKQGDYDEAIEYYQKALELDPNNAEARKNLIIADLKQEDIHHHHHH  MSELGEALILAAERGKKDRVKDLIEEGADVNASDSDGRTPLHHAAENGHAEVVALLIEKGADVNAKDSDGRTPLHHAAENGHDEVVLILLLKGADVNAKDSDGRTPLHHAAENGHKRVVLVLILAGADVNTSDSDGRTPLDLAREHGNEEVVKALEKQ |
| **O32_dn3A**  **(3ltjC3_1)**  **O32_dn3B**  **(ank1C2_1)** | **MGHHHHHHGWHHHHTDPLAVILYIAILKAEKSIARAKAAEALGKIGDERAVEPLIKALKDEDALVRAAAADALGQIGDERAVEPLIEALEDEEGLVRASAAIALGQIGDERAVEPLILALADERDLVRVAAAVALGRIGDERAVEPLIVMLRDEEGEVREAAAIALGSIGGERVRAAMEELAERGRGFARKVAVNYLETHK**  **MSELGKRLIEAAENGNKKRVKDLIENGADVNASDSDGRTPLHHAAENGHAEVVALLIEKGADVNAKDSDGRTPLHHAAENGHDEVVLILLLKGADVNAKDSDGRTPLHHAAENGHKRVVLVLILAGADVNTKDEEGDTPLALALEHGNREVIKALLKQ** |
| O43_dn1A  (1na0C4_1)  O43_dn1B  (1na0C3_3) | MGTLARVAYILGAIAYAQGEYDIAITAYQVALDLDPNNAEAWYNLGNAYYKQGDYDEAIEYYQKALELDPNNAEAWYNLGNAYYKQGDYLLAIVYYAKALILDPNNAEAKQNLGNAIQKQD  MNLAEKMYKAGNAMYRKGQYTIAIIAYTLALLKDPNNAEAWYNLGNAAYKKGEYDEAIEAYQKALELDPNNAEAWYNLGNAYYKQGDYLEAIAYYAKALLLDPNNAEARQNLGNAMQKSELEHHHHHH |
| O43_dn2A  (1na0C4_1)  O43_dn2B  (1na0C3_3) | MGTLARVAYILGAIAYAQGEYDIAITAYQVALDLDPNNAEAWYNLGNAYYKQGDYDEAIEYYQKALELDPNNAEAWYNLGNAYYKQGDYDEAILYYVKALVLDPNNAEAKQNLGNARQKQG  MNLAEKMYKAGNAMYRKGQYTIAIIAYTLALLKDPNNAEAWYNLGNAAYKKGEYDEAIEAYQKALELDPNNAEAWYNLGNAYYKQGDYLEAILYYVKALKLDPNNAEAKQNLGNAEQKKDLEHHHHHH |
| O43_dn3A  (1na0C4_1)  O43_dn3B  (1na0C3_7) | MGTLARVAYILGAIAYAQGEYDIAITAYQVALDLDPNNAEAWYNLGNAYYKQGDYDEAIKYYQKALELDPNNAEAWYNLGNAYYKQGDYVIAIALYQLALELDPNNAEAKQNLGNAEQKEGDIHHHHHH  MNSAEAMYKMGNAAYKQGDYILAIIAYLLALEKDPNNAEAWYNLGNAAYKQGDYDEAIEYYQKALELDPNNAEAWYNLGNAYYKQGDYLEAIEYYIKALELDPNNEEARQNLLNAAKKIE |
| **O43_dn4A**  **(1na0C4_1)**  **O43_dn4B**  **(1na0C3_7)** | **MGTLARVAYILGAIAYAQGEYDIAITAYQVALDLDPNNAEAWYNLGNAYYKQGDYDEAIEYYQKALELDPNNAEAWYNLGNAYYKQGDYEEAIEYYLKALELDPNNAEARQNLRNAMQKEG**  **MNSAEAMYKMGNAAYKQGDYILAIIAYLLALEKDPNNAEAWYNLGNAAYKQGDYDEAIEYYQKALELDPNNAEAWYNLGNAYYKQGDYLAAIIYYRRALELDPNNAEAKQNLGNAEQKEGLEHHHHHH** |
| O43_dn5A  (1na0C4_1)  O43_dn5B  (1na0C3_7) | MGTLARVAYILGAIAYAQGEYDIAITAYQVALDLDPNNAEAWYNLGNAYYKQGDYDEAIEYYQKALELDPNNAEAWYNLGNAYYKQGDYREALRYYIKALKLDPNNAEAKQNLGNALEKRG  MNSAEAMYKMGNAAYKQGDYILAIIAYLLALEKDPNNAEAWYNLGNAAYKQGDYDEAIEYYQKALELDPNNAEAWYNLGNAYYKQGDYLVAIIYYLEALELDPNNAEAKQNLGNAKQKEGLEHHHHHH |
| **O43_dn6A**  **(1na0C4_1)**  **O43_dn6B**  **(1na0C3_2)** | **MGTLARVAYILGAIAYAQGEYDIAITAYQVALDLDPNNAEAWYNLGNAYYKQGDYDEAIEYYQKALELDPNNAEAWYNLGNAYYKQGDYQEAIEYYARALRRDRRNKEAIENLINALQKED**  **MEEAELAYLLGELAYKLGEYRIAIRAYRIALKRDPNNAEAWYNLGNAYYKQGRYVRALIYYLRALLLDPENAEAWYNLGNAYYKKGDYDIAIVYYELALEDDPNNAEAKQLLGNAKQKQGLEHHHHHH** |
| O43_dn7A  (1na0C4_1)  O43_dn7B  (3ltjC3_1) | MGTLARVAYILGAIAYAQGEYDIAITAYQVALDLDPNNAEAWYNLGNAYYKQGDYDEAIEYYQKALELDPNNAEAWYNLGNAYYKQGDYDEAIEYYKKALRLDPNNEEAKQNLMNALQKQD  MHHHHTDPLAVILYIAILKAEKSIARAKAAEALGKIGDERAVEPLIKALKDEDALVRAAAADALGQIGDERAVEPLIKALKDEEGLVRASAAIALGQIGDERAVEPLIKALKDERDLVRVAAAVALGRIGDKKAVLPLIKALKDEEGEVREAAAIALGSIGGRLVRAMMELLAETGRGFARKVAVNYLETHKLEHHHHHH |
| O43_dn8A  (HR04C4_1)  O43_dn8B  (1na0C3_3) | MGDECEEKARLLAELVETLKRSGTSEDEIAEDVARLISEMIRNLKESGSSYEVICECVARIVAEIVEALKRSGTSAVEIAKIVARVISEVIRTLKESGSSYEVICECVARIVAEIVEALKRSGTSAAIIALIVALVISEVIRTLKESGSSFEVILECVIRIVLEIIEALKRSGTSEQDVMLIVMAVLLVVLATLQLSGS  MNLAEKMYKAGNAMYRKGQYTIAIIAYTLALLKDPNNAEAWYNLGNAAYKKGEYDEAIEAYQKALELEPNNAEAWYNLGNAYYKQGDYEEAIIYYLKALVLDPRNAEARQNLGNAKQKEGLEHHHHHH |
| O43_dn9A  (HR04C4_1)  O43_dn9B  (1na0C3_3) | MGDECEELARIVAELVEKLKRSGTSEDEIAERVAREISEVIKLLKKSGSSYEVICECVARIVAEIVEALKRSGTSAVEIAKIVARVISEVIRTLKESGSSYEVICECVARIVAEIVEALKRSGTSAAIIALIVALVISEVIRTLKESGSSFEVILECVIRIVLEIIEALKRSGTSEQDVMLIVMAVLLVVLATLQLSGS  MNLAEKMYKAGNAMYRKGQYTIAIIAYTLALLKDPNNAEAWYNLGNAAYKKGEYDEAIEAYQKALELDPENAEAWYNLGNAYYKQGEYLEALLYYLKALILDPNNAEAKQNLGNARQKQGLEHHHHHH |
| O43_dn10A  (HR04C4_1)  O43_dn10B  (1na0C3_3) | MGDECERKARLVAKIVELLKRSGTSEDEIAEEVARLISLVIKVLKKSGSSYEVICECVARIVAEIVEALKRSGTSAVEIAKIVARVISEVIRTLKESGSSYEVICECVARIVAEIVEALKRSGTSAAIIALIVALVISEVIRTLKESGSSFEVILECVIRIVLEIIEALKRSGTSEQDVMLIVMAVLLVVLATLQLSGS  MNLAEKMYKAGNAMYRKGQYTIAIIAYTLALLKDPNNAEAWYNLGNAAYKKGEYDEAIEAYQKALELDPENAEAWYNLGNAYYKQGDYAEAMLYYLKALLLDPNNAEAKQNLGNAEQKAGLEHHHHHH |
| O43_dn11A  (HR04C4_1)  O43_dn11B  (1na0C3_7) | MGDECEEKAELVALLVEALKKLGTSEDEIAEEVAKEISRVIRRLKESGSSYEVICECVARIVAEIVEALKRSGTSAVEIAKIVARVISEVIRTLKESGSSYEVICECVARIVAEIVEALKRSGTSAAIIALIVALVISEVIRTLKESGSSFEVILECVIRIVLEIIEALKRSGTSEQDVMLIVMAVLLVVLATLQLSGS  MNSAEAMYKMGNAAYKQGDYILAIIAYLLALEKDPNNAEAWYNLGNAAYKQGDYDEAIEYYQKALELDPENAEAWYNLGNAYYKQGDYELAIIFYKVALALDPNNAEAKQNLGNAKQKQGLEHHHHHH |
| **O43_dn12A**  **(HR04C4_1)**  **O43_dn12B**  **(1na0C3_2)** | **MGDRCERRAKLVALKVELLKKDGTSEDEIAEEVAREISEVIRDLRKSGSSYEVICECVARIVAEIVEALKRSGTSAVEIAKIVARVISEVIRTLKESGSSYEVICECVARIVAEIVEALKRSGTSAAIIALIVALVISEVIRTLKESGSSFEVILECVIRIVLEIIEALKRSGTSEQDVMLIVMAVLLVVLATLQLSGS**  **MEEAELAYLLGELAYKLGEYRIAIRAYRIALKRDPNNAEAWYNLGNAYYKQGDYDEAIEYYQKALELDPNNAEAWYNLGNAYYKQGDYDEAIEYYQKALELDPNNIKAELNLIIAEEKQGLEHHHHHH** |
| O43_dn13A  (HR04C4_1)  O43_dn13B  (1na0C3_2) | MGDECEELARAVALVVEILKRSGTSEDEIAEEVARLISRVIRKLKESGSSYEVICECVARIVAEIVEALKRSGTSAVEIAKIVARVISEVIRTLKESGSSYEVICECVARIVAEIVEALKRSGTSAAIIALIVALVISEVIRTLKESGSSFEVILECVIRIVLEIIEALKRSGTSEQDVMLIVMAVLLVVLATLQLSGS  MEEAELAYLLGELAYKLGEYRIAIRAYRIALKRDPNNAEAWYNLGNAYYKQGDYDEAIEYYQKALELDPNNAEAWYNLGNAYYKKGDYLIAILYYLVALTLDPNNAEAKQNLGNAKQKDGLEHHHHHH |
| O43_dn14A  (HR04C4_1)  O43_dn14B  (1na0C3_2) | MGDKCEEMAELVAQLVELLKESGTSEDEIAEKVARLISKVIRKLKESGSSYEVICECVARIVAEIVEALKRSGTSAVEIAKIVARVISEVIRTLKESGSSYEVICECVARIVAEIVEALKRSGTSAAIIALIVALVISEVIRTLKESGSSFEVILECVIRIVLEIIEALKRSGTSEQDVMLIVMAVLLVVLATLQLSGS  MEEAELAYLLGELAYKLGEYRIAIRAYRIALKRDPNNAEAWYNLGNAYYKQGDYDEAIEYYMKALKLDPKNAEAWYNLGNAYYKQGDYLLAILIYEMALILDPNNAEAKQNLGNAKQKEGLEHHHHHH |
| O43_dn15A  (HR04C4_1)  O43_dn15B  (1na0C3_2) | MGRKCELLARLVAMIVELLKESGTSEDEIAEEVAREISEVIRTLKEEGSSYEVICECVARIVAEIVEALKRSGTSAVEIAKIVARVISEVIRTLKESGSSYEVICECVARIVAEIVEALKRSGTSAAIIALIVALVISEVIRTLKESGSSFEVILECVIRIVLEIIEALKRSGTSEQDVMLIVMAVLLVVLATLQLSGS  MEEAELAYLLGELAYKLGEYRIAIRAYRIALKRDPNNAEAWYNLGNAYYKQGDYDEAIEYYQKALELDPRNAEAWYNLGNAYYKQGDYLMAILIYQLALMLDPNNAEAKQNLGNAKQKRGLEHHHHHH |
| O43_dn16A  (HR04C4_1)  O43_dn16B  (1na0C3_2) | MGEDCEELAELVAELVERLKRRGTSEDEIAEEVARIISEVIRMLKESGSSYEVICECVARIVAEIVEALKRSGTSAVEIAKIVARVISEVIRTLKESGSSYEVICECVARIVAEIVEALKRSGTSAAIIALIVALVISEVIRTLKESGSSFEVILECVIRIVLEIIEALKRSGTSEQDVMLIVMAVLLVVLATLQLSGS  MEEAELAYLLGELAYKLGEYRIAIRAYRIALKRDPNNAEAWYNLGNAYYKQGDYKEAIKYYQKALKLDPNNAEAWYNLGNAYYKKGDYIMAILAYELALEEDPNNAEAKQNLGNAKQKQGLEHHHHHH |
| **O43_dn17A**  **(HR04C4_1)**  **O43_dn17B**  **(1na0C3_2)** | **MGDECEEKARRVALKVLKLRLRGTSEDEIAEEVAREISKVIETLKESGSSYEVICECVARIVAEIVEALKRSGTSAVEIAKIVARVISEVIRTLKESGSSYEVICECVARIVAEIVEALKRSGTSAAIIALIVALVISEVIRTLKESGSSFEVILECVIRIVLEIIEALKRSGTSEQDVMLIVMAVLLVVLATLQLSGS**  **MEEAELAYLLGELAYKLGEYRIAIRAYRIALKRDPNNAEAWYNLGNAYYKQGDYDEAIEYYQKALELDPNNAEAWYNLGNAYYKQGDYDEAIEYYQKALELDPNNEEAKIVLGLAKEEQELEHHHHHH** |
| **O43_dn18A**  **(HR04C4_1)**  **O43_dn18B**  **(1na0C3_2)** | **MDRCEELARRIAEVVERAKRAGTSEDEIAESVARVISLVIRALKLSGSSYEVICECVARIVAEIVEALKRSGTSAVEIAKIVARVISEVIRTLKESGSSYEVICECVARIVAEIVEALKRSGTSAAIIALIVALVISEVIRTLKESGSSFEVILECVIRIVLEIIEALKRSGTSEQDVMLIVMAVLLVVLATLQLSGSGGWLEHHHHHH**  **MGEEAELAYLLGELAYKLGEYRIAIRAYRIALKRDPNNAEAWYNLGNAYYKQGDYDEAIEYYQKALELDPNNAEAWYNLGNAYYKQGDYDEAIEYYQKALELDPSNLDAAVNLGAATMLTS** |
| **I32_dn1A**  **(1na0C3_3)**  **I32_dn1B**  **(ank1C2_1)** | **MGNLAEKMYKAGNAMYRKGQYTIAIIAYTLALLKDPNNAEAWYNLGNAAYKKGEYDEAIEAYQKALELEPENAEALYNLGNAYYKQGEYDEAILYYLIALELDPNNAEAKQNLGNAKQKQGDIHHHHHH**  **MSRLGIRLIIAAIEGNKDRVKDLIENGADVNASDSVGRTPLHHAAENGHAEVVALLIEKGADVNAKDSDGRTPLHHAAENGHDEVVLILLLKGADVNAKDRDGRTPLHHAAENGHKRVVLVLILAGADVNTSDSDGRTPLDLAREHGNEEVVKALEKQ** |
| **I32_dn2A**  **(1na0C3_3)**  **I32_dn2B**  **(ank1C2_1)** | **MGDLAEKMYKAGNAMYRKGQYTIAIIAYTLALLKDPNNAEAWYNLGNAAYKKGEYDEAILAYLKALELDPNNAEAWYNLGNAFYKQGDYRMAIKYYQKALELDPNNAEAKQNLGNAKQKQG**  **MSELGELLIVAAENGNKKMVRDLIKNGADVNASDEDGRTPLHHAAENGHAEVVALLIEKGADVNAKDSDGRTPLHHAAENGHDEVVLILLLKGADVNAKDSDGRTPLHHAAENGHKRVVLVLILAGADVNTSDSDGRTPLDLAREHGNEEVVKALEKQLEHHHHHH** |
| I32_dn3A  (1na0C3_3)  I32_dn3B  (ank1C2_1) | MGRLAKKMYKAGNAMYRKGQYTIAIIAYTLALLKDPNNAEAWYNLGNAAYKKGEYAEAIVAYIKALELDPNNAEAWYNLGNALYKLGAYNAAIQVYQKALELDPNNAEAKQNLGNAKQKKG  MKILGLALIAAARNGEKERVETLIEAGADVNASDDDGRTPLHHAAENGHAEVVALLIEKGADVNAKDSDGRTPLHHAAENGHDEVVLILLLKGADVNAKDSDGRTPLHHAAENGHKRVVLVLILAGADVNTSDSDGRTPLDLAREHGNEEVVKALEKQLEHHHHHH |
| I32_dn4A  (1na0C3_7)  I32_dn4B  (ank1C2_1) | MGKSAEAMYKMGNAAYKQGDYILAIIAYLLALEKDPKNAEAWYNLGNAAYKQGDYEEAIRYYLKALLLDDNNAEAWYNLGNAYYKQGDYREAIMLYQKALELDPNNAEAKQNLGNAKQKQG  MSELGKLLIMAAELGNKRLVKELIENGADVNASDSDGRTPLHHAAENGHAEVVALLIEKGADVNAKDSDGRTPLHHAAEEGHDEVVLILLLKGADVNAKDSDGRTPLHHAAENGHKRVVLVLILAGADVNTSDSDGRTPLDLAREHGNEEVVKALEKQLEHHHHHH |
| I32_dn5A  (1na0C3_7)  I32_dn5B  (ank1C2_1) | MGRSAEAMYKMGNAAYKQGDYILAIIAYLLALEKDPNNAEAWYNLGNAAYKQGDYREAIRYYLKALALDPNNAEAWYNLGNAFYKQGDYNEAIEVYQKALELDPNNAEAKQNLGNAKQKQG  MSELGRMLIEAAELGKKEIVKELIENGADVNASDSDGRTPLHHAAENGHAEVVALLIEKGADVNAKDSDGRTPLHHAAENGHDEVVLILLLKGADVNAKDSDGRTPLHHAAENGHKRVVLVLILAGADVNTSDSDGRTPLDLAREHGNEEVVKALEKQLEHHHHHH |
| **I32_dn6A**  **(1na0C3_2)**  **I32_dn6B**  **(ank1C2_1)** | **MGEEAELAYLLGELAYKLGEYRIAIRAYRIALKRDPNNAEAWYNLGNAYYKQGDYDEAIEYYQKALELDPNNAEAWYNLGNAYYKQGDYDEAIEYYQKALELDPNNDEADDNLLNADQKQDDIHHHHHH**  **MSRLGKKLIIAAERGNKDRVKDLIENGADVNASDEDGRTPLHHAAENGHAEVVALLIEKGADVNAKDSDGRTPLHHAAENGHDEVVLILLLKGADVNAKDSDGRTPLHHAAENGHKRVVLVLILAGADVNTSDSDGRTPLDLAREHGNEEVVKALEKQ** |
| **I32_dn7A**  **(1na0C3_2)**  **I32_dn7B**  **(ank1C2_1)** | **MGEEAELAYLLGELAYKLGEYRIAIRAYRIALKRDPNNAEAWYNLGNAYYKQGDYDEAIEYYQKALELDPNNAEAWYNLGNAYYKQGDYDEAIEYYQKALELDPKNMEALLDLGNAKQKQKDIHHHHHH**  **MSELGKDLIVAAALGNKDRVKDLIENGADVNASDRRGATPLHMAALNGHAEVVALLIEKGADVNAKDSDGRTPLHHAAENGHDEVVLILLLKGADVNAKDSDGRTPLHHAAENGHKRVVLVLILAGADVNTSDSDGRTPLDLAREHGNEEVVKALEKQ** |
| **I32_dn8A**  **(1na0C3_2)**  **I32_dn8B**  **(ank3C2_1)** | **MGEEAELAYLLGELAYKLGEYRIAIRAYRIALKRDPNNAEAWYNLGNAYYKQGDYDEAIEYYQKALELDPNNAEAWYNLGNAYYKQGDYLRAIAYYRKALELDPNNAEAKQNLGNAKQKIE**  **MELEGERLIEAAENGNKDRVKDLLENGALVNASDSDGKTPLHLAAENGHAKVVLLLLEQGAKPNAKDSDGKTPLHLAAENGHAVVVALLLMHGADPNAKDSDGKTPLHLAAENGHEEVVILLLAMGADPNTSDSDGRTPLDLAREHGNEEVVKVLEDHGGWLEHHHHALEHHHHHH** |
| I32_dn9A  (HR00C3_2)  I32_dn9B  (ank1C2_1) | MGIEEVVAEMIDILAESSKKSIEELARAADNKTTEKAVAEAIEEIARLATAAIQLIEALAKNLASEEFMADAISAIAELAKKAIEAIYRLADNHTTDTFMARAIAAIANLAVTAILAIAALASNHTTEQFMAIAIIAIAELAKKAIEAIYRLADNHTTDKFMAAAIEAIALLATLAILAIALLASNHTTEAFMALAILLIAELAKKAIEAIYRLADNHTSPTYIEKAIEAIEKIARKAIKAIEMLAKNITTEEYKEKARAAILEIREKAKEAIKRLEDNRT  MHHHHHHSELGKRLIEAAENGNKKRVLELIENGADVNASDSDGRTPLHHAAENGHAEVVALLIELGADVNAKDSDGRTPLHHAAENGHDEVVLILLLKGADVNAKDSDGRTPLHHAAENGHKRVVLVLILAGADVETSDSRGRTPLMLAVEHGNIEVALALLKQGW |
| **I32_dn10A**  **(HR00C3_2)**  **I32_dn10B**  **(ank1C2_1)** | **MGHHHHHHWGIEEVVAEMIDILAESSKKSIEELARAADNKTTEKAVAEAIEEIARLATAAIQLIEALAKNLASEEFMARAISAIAELAKKAIEAIYRLADNHTTDTFMARAIAAIANLAVTAILAIAALASNHTTEEFMARAISAIAELAKKAIEAIYRLADNHTTDKFMAAAIEAIALLATLAILAIALLASNHTTEEFMAKAISAIARLAKKAILAIYKLADNHTSPTYIEKAIEAIEKIARKAIKAIEMLAKNITTEEYKEKAKSAIDEIREIAKIAIKTLEDNRT**  **MSEIGKRLIEAAENGNKERVKLLIELGADVNASDSDGRTPLHHAAENGHAEVVALLIEKGADVNAKDSDGRTPLHHAAENGHDEVVLILLLKGADVNAKDSDGRTPLHHAAENGHKRVVLVLILAGADVNTSDSDGRTPLDLAREHGNEEVVKALEKQ** |
| I53_dn1A  (2B98)  I53_dn1B  (HR00C3_2) | MGHHHHHHKKVGIVDTTFARVDMAIMAIIVLELRPRNIKIIRKTVPGIKDLPVACKKLLEEEGCDIVMALGMPGKAEKDKVCAHEASLGLMLAQLMTNKHIIEVFVHEDEAKDDRELDWLAKRRAEEHAENVYYLLFKPEYLTEMAGKGLRQGFEDAGP  MIEEVVAEMIDILAESSKKSIRELAKAAKNKTTEKAVAEAIEEIARLATAAIQLIEALAKNLASEEFMARAISAIAELAKKAIEAIYRLADKHKTDTFMARAIAAIANLAVTAILAIAALASNHTTEEFMARAISAIAELAKKAIMAILLLALLHTTDKFMAAAIEAIALLATLAILAIALLASNHTTEEFMAKAISAIAELAKKAIEAIYLLADLHTPVLYEDKAIEAIEKIARKAIKAIEMLAKNITTEEYKEKAKSAIDEIREKAKEAIKRLERNRE |
| I53_dn2A  (2JFB)  I53_dn2B  (1na0C3_2) | MGRYDGSKLRIGILHARWNRSIILALVLGAIERLLEFGVRAKNIIIETVPGSFELPYGSKLFVEKQKRLGKPLDAIIPIGVLIKGSTMHFEYICDSTTHQLMKLNFELGIPVIFGVLTCLTDEQAEARAGLIDGKMHNHGEDWGAAAVEMATKFN  MEEAELAYLLGELAYKLGEYRIAIRAYRIALKRDPNNAEAWYNLGNAYYKQGDYDEAIEYYRRALKLEPENAEAWYNLGNAYYKQGDYKEAIAYYLIALILDPNNAEAKQNLGNAEQKQDLEHHHHHH |
| I53_dn3A  (2JFB)  I53_dn3B  (1na0C3_2) | MGKYDGSKLRIGILHARWNRAIIIALVLGALKRLLEFGVKAKNIIIETVPGSFELPYGSKLFVEKQKRLGKPLDAIIPIGVLIKGSTMHFEYICDSTTHQLMKLNFELGIPVIFGVLTCLTDEQAEARAGLIKGKMHNHGEDWGAAAVEMATKFN  MEEAELAYLLGELAYKLGEYRIAIRAYRIALKRDPNNAEAWYNLGNAYYKQGDYDEAIEYYQEALELDPENAEAWYNLGNAYYKQGDYKEALAYYLLALELDPNNAEAEQNLGNAEQKRDLEHHHHHH |
| I53_dn4A  (2JFB)  I53_dn4B  (1na0C3_2) | MGKYDGSKLRIGILHARWNVKIIIALILGAIKRLREFGVKRENIIIEIVPGSFELPYGSKLFVEKQKRLGKPLDAIIPIGVLIKGSTMHFEYICDSTTHQLMKLNFELGIPVIFGVLTCLTDEQAEARAGLIEGKMHNHGEDWGAAAVEMATKFN  MEEAELAYLLGELAYKLGEYRIAIRAYRIALKRDPNNAEAWYNLGNAYYKQGDYDEAIEYYQKALELDPNNAEAWYNLGNAYYKQGDYDEAIEYYQKALELDPNNLDAVMNLLEASLKQELEHHHHHH |
| **I53_dn5A**  **(2JFB)**  **I53_dn5A.1 (2JFB)**  **I53_dn5B**  **(1na0C3_2)** | **MGKYDGSKLRIGILHARWNAEIILALVLGALKRLQEFGVKRENIIIETVPGSFELPYGSKLFVEKQKRLGKPLDAIIPIGVLIKGSTMHFEYICDSTTHQLMKLNFELGIPVIFGVLTCLTDEQAEARAGLIEGKMHNHGEDWGAAAVEMATKFN(LEHHHHHH)**  **MGKYDGSKLRIGILHARGNAEIILALVLGALKRLQEFGVKRENIIIETVPGSFELPYGSKLFVEKQKRLGKPLDAIIPIGVLIRGSTPHFDYIADSTTHQLMKLNFELGIPVIFGVITADTDEQAEARAGLIEGKMHNHGEDWGAAAVEMATKFN(LEHHHHHH)**  **MEEAELAYLLGELAYKLGEYRIAIRAYRIALKRDPNNAEAWYNLGNAYYKQGRYREAIEYYQKALELDPNNAEAWYNLGNAYYERGEYEEAIEYYRKALRLDPNNADAMQNLLNAKMREELEHHHHHH** |
| **I53_dn6A**  **(2JFB)**  **I53_dn6B**  **(1na0C3_2)** | **MGDYDGSKLRIGILHARKNTEIIVALVIGAVERLEEFGVKRENIIIEIVPGSFELPYGSKLFVEKQKRLGKPLDAIIPIGVLIKGSTMHFEYICDSTTHQLMKLNFELGIPVIFGVLTCLTDEQAEARAGLIEGKMHNHGEDWGAAAVEMATKFN**  **MEEAELAYLLGELAYKLGEYRIAIRAYRIALKRDPNNAEAWYNLGNAYYKQGDYDEAIEYYQKALELDPNNAEAWYNLGNAYYKQGDYDEAIEYYKKALRLDPDNAKALLNLIEAILKQKLEHHHHHH** |
| I53_dn7A  (2JFB)  I53_dn7B  (3ltjC3_1) | MGKYDGSKLRIGILHARWNRRIILALVIGAIIRLLEFGVKEDNIIIETVPGSFELPYGSKLFVEKQKRLGKPLDAIIPIGVLIKGSTMHFEYICDSTTHQLMKLNFELGIPVIFGVLTCLTDEQAEARAGLIEGKMHNHGEDWGAAAVEMATKFN  MHHHHTDPLAVILYIAILKAEKSIARAKAAEALGKIGDERAVEPLIKALKDEDALVRAAAADALGQIGDERAVIPLLRALLDKEGLVRASAAIALGQIGDKRAVLILILALEDERDLVRVAAAVALGRIGDEKAVEPLIEALKDEEGEVREAAAIALGSIGGERVRAAMEKLAETGTGFARKVAVNYLETHKLEHHHHHH |
| **I53_dn8A**  **(2OBX)**  **I53_dn8B**  **(1na0C3_2)** | **MGHHHHHHHKDYETVRIAVVRARWHADIVRQCVMAFMKEMMRIGGRRFAVEVFDVPGAYEIPLHARTLAETGRYGAVLGTAFVVNGGIYRHEFVASAVIDGMMNVQLSTGVPVLSAVLTPHNYHDSAEHHRFFFEHFTVKGKEAARACVEILAARERI**  **MEEAELAYLLGELAYKLGEYRIAIRAYRIALKRDPNNAEAWYNLGNAYYKQGDYDEAIEYYQKALELDPNNAEAWYNLGNAYYKQGDYDEAIEYYQKALELDPENEEAIDNLLEARQKQE** |
| **I53_dn9A**  **(2OBX)**  **I53_dn9B**  **(HR00C3_2)** | **MGHHHHHHHKDYETVRIAVVRARWHAEIVDVCVLAFEIEMLDIGGDRFAVDVFDVPGAYEIPLHARTLAETGRYGAVLGTAFVVNGGIYRHEFVASAVIDGMMNVQLSTGVPVLSAVLTPHNYHDSAEHHEFFFEHFTVKGKEAARACVEILAAREKI**  **MIEEVVAEMIDILAESSKKSIEELARAADNKTTEKAVAEAIEEIARLATAAIQLIEALAKNLASEEFMARAISAIAELAKKAIEAIYRLADNHTTDTFMARAIAAIANLAVTAILAIAALASNHTTEEFMARAISAIAELAKKAIAAIYRLADNHKTDKFMAAAIEAIALLATLAILAIALLASNHTTEEFMAKAIRAIAKLAKMAILVIYALAIMHTSPTYIEKAIEAIEKIARKAIKAIEMLAKNITTEEYKEKAKSAIDEIREKAKEAIKRLEDKRE** |
| **I53_dn10A**  **(2OBX)**  **I53_dn10B**  **(HR00C3_2)** | **MGHHHHHHHKDYETVRIAVVRARWHADIVDLCVIAFELEMLLIGGRRFAVDVFDVPGAYEIPLHARTLAETGRYGAVLGTAFVVNGGIYRHEFVASAVIDGMMNVQLSTGVPVLSAVLTPHNYHDSKRHHRFFAMHFIKKGKEAARACVEILAAREKI**  **MIEEVVAEMIDILAESSKKSIEELAKAADNKTTEKAVAEAIEEIARLATAAIQLIEALAKNLASEEFMARAISAIAELAKKAIEAIYRLADNHTTDTFMARAIAAIANLAVTAILAIAALASNHTTEEFMARAISAIAELAKKAIEAILELALEHETDKFMAAAIEAIALLATLAILAIALLASNHTTEEFMAKAIEAIAQLAKLAIIAIYLLALLHESPTYIEKAIEAIEKIARKAIKAIEMLAKNITTEEYKEKAKSAIDEIREKAKEAIKRLEDKRE** |
| I53_dn11A  (2OBX)  I53_dn11B  (HR00C3_2) | MGHHHHHHHKDYETVRIAVVRARWHADIVDQCVSAFEREMAKIGGDRFAVDVFDVPGAYEIPLHARTLAETGRYGAVLGTAFVVNGGIYRHEFVASAVIDGMMNVQLSTGVPVLSAVLTPHEYHDSEIHHKIFFLLFTEKGKEAARACVEILAAREKI  MIEEVVAEMIDILAESSKKSIEELARAADNKTTEKAVAEAIEEIARLATAAIQLIEALAKNLASEEFMARAISAIAELAKKAIEAIYRLADNHTTDTFMARAIAAIANLAVTAILAIAALASNHTTEEFMARAISAIAELAKKAIEAIYRLADNHTTDKFMAAAIEAIALLATLAILAIALLASNHTTEEFMAKAISAIAELAKKAIEAIYRLADDHTSPTYIEKAIEAIEKIAKKAIKAIEMLAKNITTEEYQEKARKAILEILEKALEAIRRLEDNRR |

**Supplementary File 1B.** **Amino acid sequences for all designed two-component nanoparticles.** Sequences include initiating methionines and His_6_-tags. Designs that expressed solubly and co-eluted from IMAC are denoted in bold. Input oligomers from Supplementary File 1A are included in parentheses.

| Antigen-fused component | Sequence |
| --- | --- |
| **BG505 SOSIP.v5.2(7S)**–**T33_dn2A without N241/N289** | **MKRGLCCVLLLCGAVFVSPSQEIHARFRRGARAENLWVTVYYGVPVWKDAETTLFCASDAKAYETKKHNVWATHCCVPTDPNPQEIHLENVTEEFNMWKNNMVEQMHTDIISLWDQSLKPCVKLTPLCVTLQCTNVTNNITDDMRGELKNCSFNMTTELRDKKQKVYSLFYRLDVVQINENQGNRSNNSNKEYRLINCNTSAITQACPKVSFEPIPIHYCAPAGFAILKCKDKKFNGTGPCPSVSTVQCTHGIKPVVSTQLLLNGSLAEEEVIIRSENITNNAKNILVQFNTPVQINCTRPNNNTVKSIRIGPGQWFYYTGDIIGDIRQAHCNVSKATWNETLGKVVKQLRKHFGNNTIIRFANSSGGDLEVTTHSFNCGGEFFYCNTSGLFNSTWISNTSVQGSNSTGSNDSITLPCRIKQIINMWQRIGQAMYAPPIQGVIRCVSNITGLILTRDGGSTNSTTETFRPGGGDMRDNWRSELYKYKVVKIEPLGVAPTRCKRRVVGRRRRRRAVGIGAVSLGFLGAAGSTMGAASMTLTVQARNLLSGIVQQQSNLLRAPECQQHLLKDTHWGIKQLQARVLAVEHYLRDQQLLGIWGCSGKLICCTNVPWNSSWSNRNLSEIWDNMTWLQWDKEISNYTQIIYGLLEESQNQQEKNEQDLLELDKWASLWGSMGNLAEKMYKAGNAMYRKGQYTIAIIAYTLALLKDPNNAEAWYNLGNAAYKKGEYDEAIEAYQKALELDPNNAEAWYNLGNAYYKQGDYDEAIEYYKKALRLDPRNVDAIENLIEAEEKQGAS** |
| **BG505 SOSIP.v5.2(7S)**–**T33_dn2A** | **MKRGLCCVLLLCGAVFVSPSQEIHARFRRGARAENLWVTVYYGVPVWKDAETTLFCASDAKAYETKKHNVWATHCCVPTDPNPQEIHLENVTEEFNMWKNNMVEQMHTDIISLWDQSLKPCVKLTPLCVTLQCTNVTNNITDDMRGELKNCSFNMTTELRDKKQKVYSLFYRLDVVQINENQGNRSNNSNKEYRLINCNTSAITQACPKVSFEPIPIHYCAPAGFAILKCKDKKFNGTGPCTNVSTVQCTHGIKPVVSTQLLLNGSLAEEEVIIRSENITNNAKNILVQLNESVQINCTRPNNNTVKSIRIGPGQWFYYTGDIIGDIRQAHCNVSKATWNETLGKVVKQLRKHFGNNTIIRFANSSGGDLEVTTHSFNCGGEFFYCNTSGLFNSTWISNTSVQGSNSTGSNDSITLPCRIKQIINMWQRIGQAMYAPPIQGVIRCVSNITGLILTRDGGSTNSTTETFRPGGGDMRDNWRSELYKYKVVKIEPLGVAPTRCKRRVVGRRRRRRAVGIGAVSLGFLGAAGSTMGAASMTLTVQARNLLSGIVQQQSNLLRAPECQQHLLKDTHWGIKQLQARVLAVEHYLRDQQLLGIWGCSGKLICCTNVPWNSSWSNRNLSEIWDNMTWLQWDKEISNYTQIIYGLLEESQNQQEKNEQDLLELDKWASLWGSMGNLAEKMYKAGNAMYRKGQYTIAIIAYTLALLKDPNNAEAWYNLGNAAYKKGEYDEAIEAYQKALELDPNNAEAWYNLGNAYYKQGDYDEAIEYYKKALRLDPRNVDAIENLIEAEEKQGAS** |
| **BG505 SOSIP.v5.2(7S)**–**T33_dn10A** | **MKRGLCCVLLLCGAVFVSPSQEIHARFRRGARAENLWVTVYYGVPVWKDAETTLFCASDAKAYETKKHNVWATHCCVPTDPNPQEIHLENVTEEFNMWKNNMVEQMHTDIISLWDQSLKPCVKLTPLCVTLQCTNVTNNITDDMRGELKNCSFNMTTELRDKKQKVYSLFYRLDVVQINENQGNRSNNSNKEYRLINCNTSAITQACPKVSFEPIPIHYCAPAGFAILKCKDKKFNGTGPCTNVSTVQCTHGIKPVVSTQLLLNGSLAEEEVIIRSENITNNAKNILVQLNESVQINCTRPNNNTVKSIRIGPGQWFYYTGDIIGDIRQAHCNVSKATWNETLGKVVKQLRKHFGNNTIIRFANSSGGDLEVTTHSFNCGGEFFYCNTSGLFNSTWISNTSVQGSNSTGSNDSITLPCRIKQIINMWQRIGQAMYAPPIQGVIRCVSNITGLILTRDGGSTNSTTETFRPGGGDMRDNWRSELYKYKVVKIEPLGVAPTRCKRRVVGRRRRRRAVGIGAVSLGFLGAAGSTMGAASMTLTVQARNLLSGIVQQQSNLLRAPECQQHLLKDTHWGIKQLQARVLAVEHYLRDQQLLGIWGCSGKLICCTNVPWNSSWSNRNLSEIWDNMTWLQWDKEISNYTQIIYGLLEESQNQQEKNEQGSGSGSGSGGEEAELAYLLGELAYKLGEYRIAIRAYRIALKRDPNNAEAWYNLGNAYYKQGDYDEAIEYYQKALELDPNNAEAWYNLGNAYYKQGDYDEAIEYYEKALELDPENLEALQNLLNAMDKQG** |
| **BG505 SOSIP.v5.2(7S)**–**I53_dn5B** | **MKRGLCCVLLLCGAVFVSPSQEIHARFRRGARAENLWVTVYYGVPVWKDAETTLFCASDAKAYETKKHNVWATHCCVPTDPNPQEIHLENVTEEFNMWKNNMVEQMHTDIISLWDQSLKPCVKLTPLCVTLQCTNVTNNITDDMRGELKNCSFNMTTELRDKKQKVYSLFYRLDVVQINENQGNRSNNSNKEYRLINCNTSAITQACPKVSFEPIPIHYCAPAGFAILKCKDKKFNGTGPCTNVSTVQCTHGIKPVVSTQLLLNGSLAEEEVIIRSENITNNAKNILVQLNESVQINCTRPNNNTVKSIRIGPGQWFYYTGDIIGDIRQAHCNVSKATWNETLGKVVKQLRKHFGNNTIIRFANSSGGDLEVTTHSFNCGGEFFYCNTSGLFNSTWISNTSVQGSNSTGSNDSITLPCRIKQIINMWQRIGQAMYAPPIQGVIRCVSNITGLILTRDGGSTNSTTETFRPGGGDMRDNWRSELYKYKVVKIEPLGVAPTRCKRRVVGRRRRRRAVGIGAVSLGFLGAAGSTMGAASMTLTVQARNLLSGIVQQQSNLLRAPECQQHLLKDTHWGIKQLQARVLAVEHYLRDQQLLGIWGCSGKLICCTNVPWNSSWSNRNLSEIWDNMTWLQWDKEISNYTQIIYGLLEESQNQQEKNEQGSGSGSGSGGEEAELAYLLGELAYKLGEYRIAIRAYRIALKRDPNNAEAWYNLGNAYYKQGRYREAIEYYQKALELDPNNAEAWYNLGNAYYERGEYEEAIEYYRKALRLDPNNADAMQNLLNAKMREELEAS** |
| **HA**–**I53_dn5B** | **MKAILVVLLYTFTTANADTLCIGYHANNSTDTVDTVLEKNVTVTHSVNLLEDKHNGKLCKLRGVAPLHLGKCNIAGWILGNPECESLSTASSWSYIVETSNSDNGTCFPGDFINYEELREQLSSVSSFERFEIFPKTSSWPNHDSNKGVTAACPHAGAKSFYKNLIWLVKKGNSYPKLNQSYINDKGKEVLVLWGIHHPSTTADQQSLYQNADAYVFVGTSRYSKKFKPEIATRPKVRDQEGRMNYYWTLVEPGDKITFEATGNLVVPRYAFTMERNAGSGIIISDTPVHDCNTTCQTPEGAINTSLPFQNIHPITIGKCPKYVKSTKLRLATGLRNVPSIQSRGLFGAIAGFIEGGWTGMVDGWYGYHHQNEQGSGYAADLKSTQNAIDKITNKVNSVIEKMNTQFTAVGKEFNHLEKRIENLNKKVDDGFLDIWTYNAELLVLLENERTLDYHDSNVKNLYEKVRNQLKNNAKEIGNGCFEFYHKCDNTCMESVKNGTYDYPKYSEEAKLNREKIDGVSAEEAELAYLLGELAYKLGEYRIAIRAYRIALKRDPNNAEAWYNLGNAYYKQGRYREAIEYYQKALELDPNNAEAWYNLGNAYYERGEYEEAIEYYRKALRLDPNNADAMQNLLNAKMREEGGWELQHHHHHH** |
| **DS-Cav1**–**I53_dn5B** | **MELLILKANAITTILTAVTFCFASGQNITEEFYQSTCSAVSKGYLSALRTGWYTSVITIELSNIKENKCNGTDAKVKLIKQELDKYKNAVTELQLLMQSTPATNNRARRELPRFMNYTLNNAKKTNVTLSKKRKRRFLGFLLGVGSAIASGVAVCKVLHLEGEVNKIKSALLSTNKAVVSLSNGVSVLTFKVLDLKNYIDKQLLPILNKQSCSISNIETVIEFQQKNNRLLEITREFSVNAGVTTPVSTYMLTNSELLSLINDMPITNDQKKLMSNNVQIVRQQSYSIMCIIKEEVLAYVVQLPLYGVIDTPCWKLHTSPLCTTNTKEGSNICLTRTDRGWYCDNAGSVSFFPQAETCKVQSNRVFCDTMNSLTLPSEVNLCNVDIFNPKYDCKIMTSKTDVSSSVITSLGAIVSCYGKTKCTASNKNRGIIKTFSNGCDYVSNKGVDTVSVGNTLYYVNKQEGKSLYVKGEPIINFYDPLVFPSDEFDASISQVNEKINQSLAFIRKSDELLSAIGGSAEEAELAYLLGELAYKLGEYRIAIRAYRIALKRDPNNAEAWYNLGNAYYKQGRYREAIEYYQKALELDPNNAEAWYNLGNAYYERGEYEEAIEYYRKALRLDPNNADAMQNLLNAKMREEGGWELQHHHHHH** |

**Supplementary File 1C.** **Amino acid sequences for all antigen-fused trimeric nanoparticle components.** Sequences include initiating methionines and signal peptides.
